# Supplementary material for: Leveraging interdependencies among platform and complementors in innovation ecosystem
Source: PLoS One. 2020 Oct 5;15(10):e0239972. doi: 10.1371/journal.pone.0239972 (PMC7535022; doi:10.1371/journal.pone.0239972)
Supplement: Attachment — Submitted filename: Response toReviewers0507.doc [file pone.0239972.s002.doc]

**Original Manuscript ID:** PONE-D-19-35558

**Original Article Title: “Leveraging Interdependencies among Platform and Complementors in Innovation Ecosystem”**

**To:** PLOS ONE Editor

**Re:** Response to reviewers

Dear Editor and Reviewers,

Thank you so much for your comments and valuable advices. After reading and discussing your comments and suggestions, we carefully revised our manuscript. Some details and contents are supplemented in the revised manuscript. In the following, we try our best to clearly explain our revisions point to point.

Best regards,

Zeqiang Qian (corresponding author)

School of Economics and Management, Beijing University of Chemical Technology

**Reviewer # 1**

The paper studies the effects of collaborative innovation process in a Platform-based Innovation Ecosystem (PIE) using simulation. The topic is interesting, however the contribution of the work is not sufficient to justify publication in PLOS ONE, while several parts of the submitted paper are not clearly presented. My major comments are the following:

**Comment 1:** The paper focuses only on the co-specialization process in innovations ecosystems. However, as noted by several authors, interactions should consider the dynamically intertwined processes of co-opetition, co-evolution, and co-specialization (see for example Carayannis, 2001).

**Author response:** Thank you very much for your valuable and positive comments and they are very helpful to prove the quality of our manuscript. We have revised expression according to your comments. As the reviewer’s comments, the process of interaction between the participants involved co-opetition, co-evolution, and co-specialization. It was difficult to separate the process into a single aspect. While focusing on the co-specialization process in innovation ecosystems, this paper also shows the co-opetition between platform and complementors through simulation program, and the ultimate promotion of innovation performance also indicates the co-evolution of members of PIE. The adjustment on this part is mainly reflected in the section 1. The reference provided by the reviewer are important for us to improve the quality of this paper, we have added relevant studies of the reference to our paper. Thank you so much for your comments.

**Comment 2:** A simulation-based approach, without any empirical evidence, is not sufficient to study the aforementioned problem. Current work looks more like an academic exercise.

**Author response:** Thank you very much for your valuable and positive comments and they are very helpful to prove the quality of our manuscript. The main subjects of this paper are participants in the innovation ecosystem, including generalist complementor, specialist complementor and platform firm. It is difficult to study the innovation performance of the innovation ecosystem influenced by the interaction between participants through empirical methods.	Innovation behaviors of complementors is often unpredictable. The quantification of innovation performance has long been controversial. In addition, the main subjects of this paper does not exist in a pure form in real world ecosystems. But the types of complementary firms do have an impact on ecosystem innovation performance. As we've already shown in the new version, using NK model on the research of innovation performance can yield desirable results. Thank you so much for your comments.

**Comment 3:** The adopted model, as given in section 3, is not clearly presented. For example, every variable in equations should be rigorously defined, assumptions should be better justified, simulation parameters should be clearly defined and justified, etc. For example, the authors note that they put 100 agents randomly into the innovation performance landscape. But what “randomly” means? Do they mean "uniformly at random"? If yes, what parameters have been chosen for the uniform distribution? Why?

**Author response:** Thank you very much for your suggestions. In section 3 we've added a table to illustrate the parameters. The Sendero that we used to build our NK model can well encapsulate the parameter properties of the NK model, so that we only need to focus on the relevant parts that we need to modify, having to focus on reconstructing the whole model. The agents random distribution problem mentioned in the comment is set internally in this program. Each run of a simulation program ensures that agents are uniformly randomly distributed in the fitness landscape. Thank you for your helpful comments.

**Comment 4:** Several results are poorly discussed and justified. For example, the inverted U-shape relation is an important finding. The authors simply note this, but they do not give a suitable justification and explanation of this finding (the only justification of results can be found in lines 402-404).

**Author response:** Thank you so much for your constructive comments, which are very helpful for improving the quality of our manuscript. We have carefully analyzed your comments and made corresponding revisions. We tried to give a suitable justification and explanation of this finding. Please see the new section 5. Thank you so much for reviewing our manuscript and providing us valuable comments.

**Comment 5:** Moreover, some results seem self-evident (e.g., “…the innovation performance of complementors in PIE is affected by the complexity of the system and the dependency between innovation entities…”).

**Author response:** Thank you so much for your constructive comments, which are very helpful for improving the quality of our manuscript. We have carefully analyzed your comments and made corresponding revisions. Please see Line 501-512. Thank you so much for reviewing our manuscript and providing us valuable comments. Please see

**Comment 6:** I cannot see any significant valuable managerial implication in section 5.

**Author response:** Thank you so much for your constructive comments, which are very helpful for improving the quality of our manuscript. We have carefully analyzed your comments and made corresponding revisions. We have made a major adjustment to section 5. Thank you so much for reviewing our manuscript and providing us valuable comments.

**Reviewer # 2**

Authors proposed a novel multiple attribute decision-making method based on Schweizer-Sklar t-norm and t-conorm with q-rung dual hesitant fuzzy information. I think the paper is well written and its structure is well organized, and the study is innovative. So, I think this paper can be published in this journal by minor revisions.

**Comment 1:** 1. Fig3(a)-3(c) and Fig4(a) -4(c) are not well explained. Three dependent situations of specialist complementors and generalist complementors participating in the collaborative innovation process are vague.

**Author response:** Thank you very much for your comments, which are helpful for improving the quality of our manuscript. We have adjusted part of section 3 to reflect your comments. Please see Line 392-406. Thank you so much for your comments.

**Comment 2:** In the simulation, you should set several groups of N to get more reliable conclusions. If N is only set to be equal to 16, the conclusions may be subjective.

**Author response:** Thank you very much for your reminder. The NK model is NP-complete for K>1.When N=16, there are already 65536locations in the fitness landscape. The complexity of the model is sufficient to describe the innovation ecosystem in this paper. In addition, we conducted several sets of experiments N take other values and all obtained similar curve trends. We chose only one set of data to explain our topic from a primary and secondary perspective. Thank you so much for reviewing our manuscript.

**Comment 3:** Some words are misspelled.

**Author response:** Thank you so much for your constructive comments We have carefully checked the whole paper and revised some expressions errors in the revised manuscript. In addition, we have carefully checked the manuscript and tried our best to correct typos and linguistic errors. Thank you so much for reviewing our manuscript and providing us valuable comments.

**Reviewer # 3**

**Comment 1:** PIE performance is a key construct in this paper. But the authors fail to address how to evaluate PIE performance and existing research about it should also be included.

**Author response:** Thank you very much for your suggestions. according to your comments we have made corresponding adjustments in section 1 and 2 of the research. In this paper, the process of collaborative innovation is regarded as a search process, trial and error search process in bounded space. This process is the reorganization and development of the existing things, the change and promotion of innovation performance is an important driving force in this process, the score of each position represents the innovation output efficiency of this position component combination. The existing research about it should is included in this paper. Thank you for reviewing our manuscript.

**Comment 2:** The authors should clarify why NK model is suitable for this research.

**Author response:** Thank you very much for your suggestions. We have added some explanations to explain why the NK model is suitable for this research. Line 229-237 are the main parts that we added to illustrate this point. Thank you for reviewing our manuscript.

**Comment 3:** More latest research literature should be included in the paper.

**Author response:** Thank you very much for your suggestions. More latest research literature has been added to improve the Introduction and Section 2. Thank you for reviewing our manuscript.

**Comment 4:** The authors should revise the language to improve readability. For example, Line 20-24 are difficult to follow.

**Author response:** Thank you very much for your suggestions. We have carefully checked the whole paper and revised some expressions errors in the revised manuscript. Line 20-23 has been modified to make it easier to understand. Thank you for reviewing our manuscript.
