# Supplementary material for: Leveraging interdependencies among platform and complementors in innovation ecosystem
Source: PLoS One. 2020 Oct 5;15(10):e0239972. doi: 10.1371/journal.pone.0239972 (PMC7535022; doi:10.1371/journal.pone.0239972)
Supplement: Attachment — Submitted filename: Response toReviewers0731.doc [file pone.0239972.s003.doc]

**Original Manuscript ID:** PONE-D-19-35558

**Original Article Title: “Leveraging Interdependencies among Platform and Complementors in Innovation Ecosystem”**

**To:** PLOS ONE Editor

**Re:** Response to reviewers

Dear Editor and Reviewers,

Thank you so much for your comments and valuable advices. After a second reading and discussing your comments and suggestions, we carefully revised our manuscript. Some details and contents are supplemented in the revised manuscript. In the following, we try our best to clearly explain our revisions point to point.

Best regards,

Zeqiang Qian (corresponding author)

School of Economics and Management, Beijing University of Chemical Technology

**Reviewer # 1**

The revised version of the paper has considered some of my previous comments, however my major concerns have not appropriately addressed:

**Comment 1:** In my previous report I asked for empirical evidence about the presented simulation-based approach. The authors, in their response, note that this difficult and innovation behavior is often unpredictable. I can understand this difficulty, I can understand that it is not possible to have extensive empirical data, but at least some empirical evidence should be given. Otherwise, this is an excellent academic exercise, but with little usefulness.

**Author response:** Thank you very much for your valuable and positive comments and they are very helpful to prove the quality of our manuscript. We have revised expression according to your comments. We added two study cases as empirical evidence. The adjustment on this part is mainly reflected in the section 4. Thank you so much for your comments.

**Comment 2:** As noted in my previous report, the model should be clearly presented (parameters, variables, equations, etc.). This is still unclear in the revised text. See for example the very first equation: F is the fitness of the system, but fi, ni, n have not been defined.

**Author response:** Thank you very much for your valuable and positive comments and they are very helpful to prove the quality of our manuscript. The adjustment on this part is mainly reflected in the section 3. Please see Line 249-264. Thank you so much for your comments.

**Comment 3:** Similarly, the assumptions of the simulation model seem arbitrary, I cannot see any justification in the revised manuscript, although I asked for this in my previous report.

**Author response:** Thank you very much for your suggestions. In section 3 we've adjusted assumptions. We also We rethought your previous suggestion. It is very helpful to us. Please see Line 403-407. Thank you for your helpful comments.

**Reviewer # 2**

**Comment :** The manuscript is much better. But I think there are some problems. As you said, when N=16, there are already 65536 locations in the fitness landscape, the complexity of the model is sufficient to describe the innovation ecosystem in this paper. I think you should give a sufficient reason why do not you choose N=14 or N=18. You should have more evidence why N=16 is the best choise. If you did other experients, plesse list them.

**Author response:** Thank you very much for your comments, which are helpful for improving the quality of our manuscript. The aim of our study is to employ the NK model of complex evolving systems to analyze/explore the collaborative innovation processes of the innovation ecosystem. According to previous studies(see for example Baumann, 2019), the NK model is a stochastic combinatorial optimization model with two parameters (N, K) for studying innovation. In our study, N is the components of the innovation ecosystem. Parameters N represent the size of the simulation object. Parameter N is a basic parameter. We need to focus on parameter K and the distribution of K. To sum up, considering the scale of the simulation experiment and the length of the paper, we set the parameter N=16.Thank you so much for your comments.

Reference:

Baumann O, Schmidt J, Stieglitz N. Effective search in rugged performance landscapes: A review and outlook. Journal of Management. 2019 Jan;45(1):285-318.

**Reviewer # 3**

**Comment 1:** There are some long sentences which maybe hard to understand. I suggest that the author should shorten these long sentences. e.g. Line 66-68.

**Author response:** Thank you very much for your suggestions. We have adjusted the parts. Thank you for reviewing our manuscript.

**Comment 2:** Line 12, the word "However" can be changed with "Although". In Line 118," in level of" can be change with" in forms of ".

**Author response:** Thank you very much for your suggestions. We have adjusted the two parts. Thank you for reviewing our manuscript.

**Comment 3:** The method of this manuscript is well described.

**Author response:** Thank you very much for your recognition. This greatly encouraged us to continue our academic research. Thank you for reviewing our manuscript.

**Comment 4:** The research gap is not clear enough.

**Author response:** Thank you very much for your suggestions. The adjustment on this part is mainly reflected in the section 2 and section 4. The references have also been adjusted. Thank you for reviewing our manuscript.
