# Supplementary material for: Leveraging interdependencies among platform and complementors in innovation ecosystem
Source: PLoS One. 2020 Oct 5;15(10):e0239972. doi: 10.1371/journal.pone.0239972 (PMC7535022; doi:10.1371/journal.pone.0239972)
Supplement: Attachment — Submitted filename: Response toReviewers0912.doc [file pone.0239972.s004.doc]

**Original Manuscript ID:** PONE-D-19-35558

**Original Article Title: “Leveraging Interdependencies among Platform and Complementors in Innovation Ecosystem”**

**To:** PLOS ONE Editor

**Re:** Response to reviewers

Dear Editor and Reviewers,

Thank you so much for your comments and valuable advices. After reading and discussing your comments and suggestions, we carefully revised our manuscript. Some details and contents are supplemented in the revised manuscript. In the following, we try our best to clearly explain our revisions point to point.

Best regards,

Zeqiang Qian (corresponding author)

School of Economics and Management, Beijing University of Chemical Technology

**Reviewer # 2**

**Comment :** I can not see any revisions about my previous comments in this manuscript.You do not give the sufficient reason that you choose N=16. The choice of N is arbitrary. In NK model 0<=K<=N-1, if you do not choose appropriate N, K may be not appropriate. Thus, your simulation and conclusions may be wrong. You should give sufficient envidences about the choice of N.

**Author response:** Thank you very much for your comments, which are helpful for improving the quality of our manuscript. Our simulation results do need to avoid subjective. under our existing simulation experiment environment, we set the maximum of N and rerun the simulation program. The adjustment on this part is mainly reflected in the section 4. Adjustments based on your comments do lead to get more reliable conclusions. Thank you so much for your comments.

**Reviewer # 3**

**Comment :** This paper uses improved NK model to simulate innovation process between platform firms and completmentors. This is a good method and the autors described the method very clearly. But I find some sentences difficult to understand; for example, LINE 12-15, LINE 28-29. Please rewrite these sentences.

**Author response:** Thank you very much for your suggestions. We have adjusted the two parts. Thank you for reviewing our manuscript.

**Author response:** Thank you very much for your suggestions. The adjustment on this part is mainly reflected in the section 2 and section 4. The references have also been adjusted. Thank you for reviewing our manuscript.
